# Reproductive profiles and risk of breast cancer subtypes: a multi-center case-only study

**DOI:** 10.1186/s13058-017-0909-3

**Published:** 2017-11-07

**Authors:** Olivier Brouckaert, Anja Rudolph, Annouschka Laenen, Renske Keeman, Manjeet K. Bolla, Qin Wang, Adelheid Soubry, Hans Wildiers, Irene L. Andrulis, Volker Arndt, Matthias W. Beckmann, Javier Benitez, Carl Blomqvist, Stig E. Bojesen, Hiltrud Brauch, Paul Brennan, Hermann Brenner, Georgia Chenevix-Trench, Ji-Yeob Choi, Sten Cornelissen, Fergus J. Couch, Angela Cox, Simon S. Cross, Kamila Czene, Mikael Eriksson, Peter A. Fasching, Jonine Figueroa, Henrik Flyger, Graham G. Giles, Anna González-Neira, Pascal Guénel, Per Hall, Antoinette Hollestelle, John L. Hopper, Hidemi Ito, Michael Jones, Daehee Kang, kConFab, Julia A. Knight, Veli-Matti Kosma, Jingmei Li, Annika Lindblom, Jenna Lilyquist, Artitaya Lophatananon, Arto Mannermaa, Siranoush Manoukian, Sara Margolin, Keitaro Matsuo, Kenneth Muir, Heli Nevanlinna, Paolo Peterlongo, Katri Pylkäs, Suleeporn Saajrang, Caroline Seynaeve, Chen-Yang Shen, Xiao-Ou Shu, Melissa C. Southey, Anthony Swerdlow, Soo-Hwang Teo, Rob A. E. M. Tollenaar, Thérèse Truong, Chiu-chen Tseng, Alexandra J. van den Broek, Carolien H. M. van Deurzen, Robert Winqvist, Anna H. Wu, Cheng Har Yip, Jyh-Cherng Yu, Wei Zheng, Roger L. Milne, Paul D. P. Pharoah, Douglas F. Easton, Marjanka K. Schmidt, Montserrat Garcia-Closas, Jenny Chang-Claude, Diether Lambrechts, Patrick Neven

**Affiliations:** 1 Department of Obstetrics and Gynaecology, Jan Yperman Hospital, Ypres, Belgium; 2 Division of Cancer Epidemiology, German Cancer Research Center (DKFZ), Heidelberg, Germany; 3 Centre for Biostatistics and Statistical Bioinformatics, KU Leuven, Leuven, Belgium; 4 Netherlands Cancer Institute, Antoni van Leeuwenhoek hospital, Amsterdam, The Netherlands; 5 Centre for Cancer Genetic Epidemiology, Department of Public Health and Primary Care University of Cambridge, Cambridge, UK; 6 Epidemiology Research Unit, Department of Public Health and Primary Care, Faculty of Medicine, KU Leuven - University of Leuven, Leuven, Belgium; 7 Department of Oncology, Leuven Multidisciplinary Breast Cancer, University Hospital Leuven, KU Leuven, Leuven, Belgium; 8 Lunenfeld-Tanenbaum Research Institute of Mount Sinai Hospital, Toronto, Canada; 9 Department of Molecular Genetics, University of Toronto, Toronto, Canada; 10 Division of Clinical Epidemiology and Aging Research, German Cancer Research Center (DKFZ), Heidelberg, Germany; 11 Department of Gynaecology and Obstetrics, University Hospital Erlangen, Friedrich-Alexander University Erlangen-Nuremberg, Comprehensive Cancer Center Erlangen-EMN, Erlangen, Germany; 12 Human Cancer Genetics Program, Spanish National Cancer Research Centre, Madrid, Spain; 13 Centro de Investigación en Red de Enfermedades Raras, Valencia, Spain; 14 Department of Oncology, Helsinki University Hospital, University of Helsinki, Helsinki, Finland; 15 Copenhagen General Population Study, Herlev and Gentofte Hospital, Copenhagen University Hospital, Herlev, Denmark; 16 Department of Clinical Biochemistry, Herlev and Gentofte Hospital, Copenhagen University Hospital, Herlev, Denmark; 17 Faculty of Health and Medical Sciences, University of Copenhagen, Copenhagen, Denmark; 18 Dr. Margarete Fischer-Bosch-Institute of Clinical Pharmacology, Stuttgart, Germany; 19 University of Tübingen, Tübingen, Germany; 20 German Cancer Consortium (DKTK), German Cancer Research Center (DKFZ), Heidelberg, Germany; 21 International Agency for Research on Cancer, Lyon, France; 22 Division of Preventive Oncology, German Cancer Research Center (DKFZ) and National Center for Tumor Diseases (NCT), Heidelberg, Germany; 23 Department of Genetics, QIMR Berghofer Medical Research Institute, Brisbane, Australia; 24 Department of Biomedical Sciences, Seoul National University College of Medicine, Seoul, Korea; 25 Cancer Research Institute, Seoul National University, Seoul, Korea; 26 Department of Laboratory Medicine and Pathology, Mayo Clinic, Rochester, MN USA; 27 Sheffield Cancer Research, Department of Oncology and Metabolism, University of Sheffield, Sheffield, UK; 28 Academic Unit of Pathology, Department of Neuroscience, University of Sheffield, Sheffield, UK; 29 Department of Medical Epidemiology and Biostatistics, Karolinska Institutet, Stockholm, Sweden; 30 David Geffen School of Medicine, Department of Medicine Division of Hematology and Oncology, University of California at Los Angeles, Los Angeles, CA USA; 31 Usher Institute of Population Health Sciences and Informatics, The University of Edinburgh Medical School, Edinburgh, UK; 32 Division of Cancer Epidemiology and Genetics, National Cancer Institute, Rockville, MD USA; 33 Department of Breast Surgery, Herlev and Gentofte Hospital, Copenhagen University Hospital, Herlev, Denmark; 34 Cancer Epidemiology & Intelligence Division, Cancer Council Victoria, Melbourne, Australia; 35 Centre for Epidemiology and Biostatistics, Melbourne School of Population and Global health, The University of Melbourne, Melbourne, Australia; 36 Cancer & Environment Group, Center for Research in Epidemiology and Population Health (CESP), INSERM, University Paris-Sud, University Paris-Saclay, Villejuif, France; 37 Department of Medical Oncology, Family Cancer Clinic, Erasmus MC Cancer Institute, Rotterdam, The Netherlands; 38 Division of Epidemiology and Prevention, Aichi Cancer Center Research Institute, Nagoya, Japan; 39 Division of Genetics and Epidemiology, The Institute of Cancer Research, London, UK; 40 Department of Preventive Medicine, Seoul National University College of Medicine, Seoul, Korea; 41 kConFab, Research Department, Peter MacCallum Cancer Centre, and The Sir Peter MacCallum Department of Oncology, University of Melbourne, Parkville, Australia; 42 Prosserman Centre for Health Research, Lunenfeld-Tanenbaum Research Institute of Mount Sinai Hospital, Toronto, Canada; 43 Division of Epidemiology, Dalla Lana School of Public Health, University of Toronto, Toronto, Canada; 44 Cancer Center of Eastern Finland, University of Eastern Finland, Kuopio, Finland; 45 Institute of Clinical Medicine, Pathology and Forensic Medicine, University of Eastern Finland, Kuopio, Finland; 46 Imaging Center, Department of Clinical Pathology, Kuopio University Hospital, Kuopio, Finland; 47 Department of Molecular Medicine and Surgery, Karolinska Institutet, Stockholm, Sweden; 48 Department of Health Sciences Research, Mayo Clinic, Rochester, MN USA; 49 Division of Health Sciences, Warwick Medical School, Warwick University, Coventry, UK; 50 Institute of Population Health, University of Manchester, Manchester, UK; 51 Unit of Medical Genetics, Department of Preventive and Predictive Medicine, Fondazione IRCCS (Istituto Di Ricovero e Cura a Carattere Scientifico) Istituto Nazionale dei Tumori (INT), Milan, Italy; 52 Department of Oncology - Pathology, Karolinska Institutet, Stockholm, Sweden; 53 Division of Molecular Medicine, Aichi Cancer Center Research Institute, Nagoya, Japan; 54 Department of Obstetrics and Gynecology, Helsinki University Hospital, University of Helsinki, Helsinki, Finland; 55 IFOM, The FIRC (Italian Foundation for Cancer Research) Institute of Molecular Oncology, Milan, Italy; 56 Laboratory of Cancer Genetics and Tumor Biology, Cancer and Translational Medicine Research Unit, Biocenter Oulu, University of Oulu, Oulu, Finland; 57 Laboratory of Cancer Genetics and Tumor Biology, Northern Finland Laboratory Centre Oulu, Oulu, Finland; 58 National Cancer Institute, Bangkok, Thailand; 59 School of Public Health, China Medical University, Taichung, Taiwan; 60 Taiwan Biobank, Institute of Biomedical Sciences, Academia Sinica, Taipei, Taiwan; 61 Division of Epidemiology, Department of Medicine, Vanderbilt-Ingram Cancer Center, Vanderbilt University School of Medicine, Nashville, TN USA; 62 Department of Pathology, The University of Melbourne, Melbourne, Australia; 63 Division of Breast Cancer Research, The Institute of Cancer Research, London, UK; 64 Cancer Research Initiatives Foundation, Subang Jaya, Selangor Malaysia; 65 Breast Cancer Research Unit, Cancer Research Institute, University Malaya Medical Centre, Kuala Lumpur, Malaysia; 66 Department of Surgery, Leiden University Medical Center, Leiden, The Netherlands; 67 Department of Preventive Medicine, Keck School of Medicine, University of Southern California, Los Angeles, CA USA; 68 Division of Psychosocial Research and Epidemiology, Netherlands Cancer Institute, Amsterdam, The Netherlands; 69 Department of Pathology, Erasmus University Medical Center, Rotterdam, The Netherlands; 70 Department of Surgery, National Taiwan University Hospital, Taipei, Taiwan; 71 Division of Epidemiology, Department of Medicine, Vanderbilt Epidemiology Center, Vanderbilt-Ingram Cancer Center, Vanderbilt University School of Medicine, Nashville, TN USA; 72 Centre for Cancer Genetic Epidemiology, Department of Oncology, University of Cambridge, Cambridge, UK; 73 University Cancer Center Hamburg (UCCH), University Medical Center Hamburg-Eppendorf, Hamburg, Germany; 74 Center for Cancer Biology, VIB, Leuven, Belgium; 75 Laboratory for Translational Genetics, Department of Human Genetics, KU Leuven, Leuven, Belgium

**Keywords:** Breast cancer subtype, Age at breast cancer diagnosis, Parity, Age at first full-time pregnancy, Age at menarche

## Abstract

**Background:**

Previous studies have shown that reproductive factors are differentially associated with breast cancer (BC) risk by subtypes. The aim of this study was to investigate associations between reproductive factors and BC subtypes, and whether these vary by age at diagnosis.

**Methods:**

We used pooled data on tumor markers (estrogen and progesterone receptor, human epidermal growth factor receptor-2 (HER2)) and reproductive risk factors (parity, age at first full-time pregnancy (FFTP) and age at menarche) from 28,095 patients with invasive BC from 34 studies participating in the Breast Cancer Association Consortium (BCAC). In a case-only analysis, we used logistic regression to assess associations between reproductive factors and BC subtype compared to luminal A tumors as a reference. The interaction between age and parity in BC subtype risk was also tested, across all ages and, because age was modeled non-linearly, specifically at ages 35, 55 and 75 years.

**Results:**

Parous women were more likely to be diagnosed with triple negative BC (TNBC) than with luminal A BC, irrespective of age (OR for parity = 1.38, 95% CI 1.16–1.65, *p* = 0.0004; *p* for interaction with age = 0.076). Parous women were also more likely to be diagnosed with luminal and non-luminal HER2-like BCs and this effect was slightly more pronounced at an early age (*p* for interaction with age = 0.037 and 0.030, respectively). For instance, women diagnosed at age 35 were 1.48 (CI 1.01–2.16) more likely to have luminal HER2-like BC than luminal A BC, while this association was not significant at age 75 (OR = 0.72, CI 0.45–1.14). While age at menarche was not significantly associated with BC subtype, increasing age at FFTP was non-linearly associated with TNBC relative to luminal A BC. An age at FFTP of 25 versus 20 years lowered the risk for TNBC (OR = 0.78, CI 0.70–0.88, *p* < 0.0001), but this effect was not apparent at a later FFTP.

**Conclusions:**

Our main findings suggest that parity is associated with TNBC across all ages at BC diagnosis, whereas the association with luminal HER2-like BC was present only for early onset BC.

**Electronic supplementary material:**

The online version of this article (doi:10.1186/s13058-017-0909-3) contains supplementary material, which is available to authorized users.

## Background

Worldwide, breast cancer (BC) is the most frequently diagnosed malignancy and leading cause of female cancer death [1]. Over the past decade, it has become evident that BC represents a heterogeneous disease, for which different subtypes can be distinguished based on the combination of tumor grade and the presence of hormone receptors, i.e., estrogen (ER), progesterone (PR) and human epidermal growth factor receptor-2 (HER2). Each BC subtype, including the luminal A-like, luminal B-like, luminal HER2-like, HER2-like and triple negative breast cancer (TNBC), presents with different age and risk factor distributions [2]. Analyses from the Breast Cancer Association Consortium (BCAC) showed, for instance, that nulliparity and a later age at first full-time pregnancy (FFTP) increase the risk of ER-positive BC, but not ER-negative BC [3].

Menarche and FFTP, and in particular their timing, may have diverse and complex effects on BC risk. It has been proposed that pregnancy induces the protective differentiation of mammary cells in the terminal duct lobular unit, which translates into long-term protection against BC [4–6]. Subsequent full-term pregnancies exert a similar but quantitatively much less important effect, which is a likely reflection of the protective differentiation of breast cells already induced by the FFTP [7]. The protective effect of an FFTP, however, is not apparent when the FFTP occurs after the age of 35 years [8, 9]. Although an FFTP offers long-term protection against BC, pregnancy is also associated with a transient increased BC risk postpartum, which could be due to pregnancy-related stimulation of pre-existent malignant clones [7, 10, 11]. In addition, the protective effect of the FFTP is found to be greater later in life [12], which according to a hypothesis by Russo et al. can be explained by the fact that several breast tumors are already initiated before the pregnancy, i.e., before the FFTP can induce its protective effect [4, 5].

Age at menarche has also been reported to influence BC risk differently depending on age. A late age at menarche is associated with a later onset of ovulatory cycles, and consequently with a decreased lifetime exposure to estrogen [13, 14]. For instance, per one year younger age at menarche, the associated BC risk increases by about 7% in women aged < 45 years, whereas the increase is about 4% in women aged 65 or older [15]. Also a short window of susceptibility, which is defined as the time between age at menarche and age at FFTP, lowers BC risk [16].

So far, few studies have examined how reproductive variables may be differentially associated with the risk of a specific BC subtype [2, 17]. The majority of these studies did not consider differential effects by age or were typically limited to cases developing BC at young age [18, 19], or to postmenopausal women only [20, 21]. Additional investigations involving women diagnosed with BC in all age categories, for which data on BC subtypes are available, are thus warranted. Therefore, we examined the association between parity and the risk of developing a specific BC subtype, and how this may differ according to age. Furthermore, we assessed the association of age at menarche (in nulliparous and parous women) and age at FFTP with the risk of being diagnosed with a specific BC subtype.

## Methods

### BCAC cohorts, inclusion and exclusion criteria

**Fig. 1:**
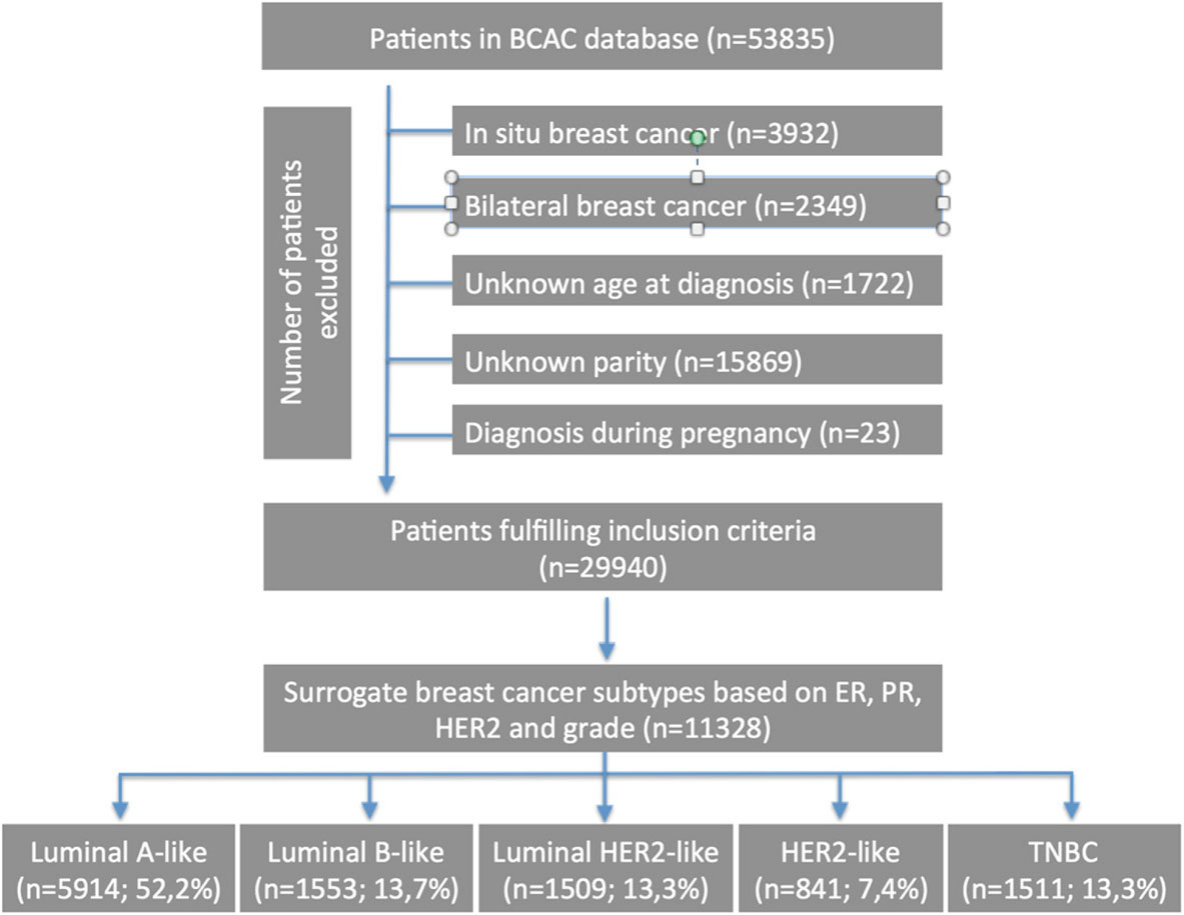
Consolidated Standards of Reporting Trials (CONSORT) diagram showing the flow of patients throughout the study. BCAC, Breast Cancer Association Consortium; HER2, human epidermal growth factor receptor-2 ; ER estrogen receptor; PR progesterone receptor; TNBC, triple negative breast cancer

This analysis includes data from studies which participate in the BCAC and could provide information on BC risk factors, in particular parity (never versus ever), age at menarche and age at FFTP, and clinic-pathological information, in particular, grade, ER, PR and HER2 status of the tumor. Studies not providing any of these data were excluded. Three additional studies in the BCAC with information on BC subtypes were also not included: two studies included only ER-negative BC or patients with TNBC (SKKDKFZS, NBCS); one study included only ER-positive/non-TNBC patients (PBCS). Overall, 34 of the 49 available studies in the BCAC provided data justifying inclusion in our study. These were composed of 10 population-based studies (8 case–control and 2 prospective cohort studies); 5 hospital-based case-control studies; and 19 studies of mixed design (all other studies). No information on previous *in situ* BC was available, though some studies excluded patients with a previous BC. Figure 1 displays the numbers of excluded and included patients. Patients with *in situ* breast cancer (N = 3932) and patients with bilateral BC (N = 2349) were excluded, since interpretation may be difficult in the case of coexistence of different BC phenotypes. Patients with BC diagnosed during pregnancy (N = 23) were excluded as well. Patients with insufficient information for assignment to BC subtype according to the 2011 St. Gallen criteria [22] (N = 18,612) were also excluded. Patients with BC before a first pregnancy were classified as nulliparous. Only BC patients with an unambiguously defined surrogate molecular BC status (N = 11,328), and with a known parity and age at BC diagnosis were included (Fig. 1).

### Tumor marker definitions and definition of breast cancer subtypes

Definitions of ER, PR and HER2 status were not standardized across studies since most data were extracted from medical records (15 of 34 studies for ER and PR, and 6 of 34 studies for HER2). The 2011 St. Gallen criteria were used to define the five surrogate BC molecular phenotypes, using grade instead of Ki67 positivity [22]: (i) luminal A-like (ER-positive and/or PR-positive, HER2-negative, grade 1 or 2), (ii) luminal B-like (ER-positive and/or PR-positive, HER2-negative, grade 3), (iii) luminal HER2-like (ER-positive and/or PR-positive and HER2-positive), (iv) HER2-like (ER-negative and PR-negative, HER2-positive), and TN (ER-negative, PR-negative and HER2-negative) BC. How these internationally accepted definitions relate to other definitions of BC subtypes, as previously also applied in other BCAC publications, is depicted in Additional file 1: Table S1.

### Statistical methodology

To evaluate the interaction between parity and age at BC diagnosis considering all BC subtypes simultaneously, a baseline-category logit model was applied to a five-category polytomous variable consisting of the five BC molecular subtypes, whereby luminal A-like breast cancer was considered the reference category. Interactions between parity and age at BC diagnosis in the probability of developing a specific BC subtype were tested by separate logistic regression models with binary outcome (1 for the BC subtype and 0 for luminal A-like BC as the reference subtype). For age at diagnosis, non-linear trends were explored by means of quadratic and cubic spline-based curves. Likelihood ratio testing was used for model selection of nested models and akaike information criterion (AIC) in the case of un-nested models. The best fit was obtained when modeling age non-linearly using cubic splines (five knots). Odds ratios (OR) with 95% confidence intervals were estimated across all ages and, since age at diagnosis was modeled non-linearly, extrapolated from the non-linear fit at three different ages: 35 years (premenopausal age), 55 years (early postmenopausal age) and 75 years (late postmenopausal age). Hence, odds ratios for parity at selected ages are deducted from the curves generated for all patients at all ages, and are not based on subgroups of patients at selected ages.

To follow up on a significant interaction between age at diagnosis (continuous) and parity in luminal HER2-like and HER2-like BC, which only differ in their ER/PR status, we tested whether this interaction varied by combined ER/PR status (ER+/PR+, ER-/PR+, ER+/PR- = 1, ER-/PR- = 0) by means of a three-way interaction test (age at diagnosis × parity × ER/PR status). The test was conducted using a binary logistic regression model (HER2 status negative/positive) with HER2-negative as the reference group. Additionally, we assessed the association between parity and HER2-positive BC by age at diagnosis, using luminal A-like BC as the reference subtype.

Associations of age at menarche (in parous and nulliparous women) and FFTP (in parous women only) with BC subtypes were evaluated using logistic regression models. Again, luminal A-like BC was used as the reference subtype. Age at BC diagnosis was included in these models to correct for possible confounding. In parous women, age at FFTP was modelled according to the best fit, i.e., as a linear function for luminal B-like, luminal HER2-like and HER2-like BC, but as a quadratic function for TNBC.

To account for clustering of patients by study, a multilevel (or random-effects) model was used to provide unbiased standard errors and *p* values [23]. All tests were two-sided and *p* values smaller than 0.05 were considered significant. The analyses were performed using SAS software, version 9.2.

## Results

Descriptive statistics for age at BC diagnosis, menopausal status, parity, age at menarche and age at FFTP stratified by molecular subtype are presented in Table 1. Additional information about these variables per individual study and for tumor size, nodal status, tumor grade and PR status can be found in Additional file 1: Tables S2-S4.

**Table 1:** Distribution of reproductive risk factors according to the five surrogate BC subtypes

		All BC subtypes N = 11,328 (100.0%)		Luminal A-like N = 5914 (52.2%)		Luminal B-like N = 1553 (13.7%)		Luminal HER2-like N = 1509 (13.3%)		HER2-like N = 841 (7.4%)		TNBC N = 1511 (13.3%)	
		N	%	N	%	N	%	N	%	N	%	N	%
Age at breast cancer diagnosis, years	Mean/median	56.0/56.0		57.6/57.0		56.9/57.0		52.6/52.0		52.9/53.0		53.8/54.0	
	<41	1077	9.5%	328	5.5%	139	9.0%	247	16.4%	120	14.3%	243	16.1%
	41–50	2759	24.4%	1369	23.1%	367	23.6%	455	30.2%	226	26.9%	342	22.6%
	51–60	51-60y	30.4%	1842	31.1%	424	27.3%	406	26.9%	287	34.1%	481	31.8%
	>60	4052	35.8%	2375	40.2%	623	40.1%	401	26.6%	208	24.7%	445	29.5%
Menopausal status	Pre/peri	3796	33.5%	1797	30.4%	505	32.5%	612	40.6%	316	37.6%	566	37.5%
	Post	7075	62.5%	3886	65.7%	979	63.0%	835	55.3%	484	57.6%	891	59.0%
	Unknown	457	4.0%	231	3.9%	69	4.4%	62	4.1%	41	4.9%	54	3.6%
Parity (>/=24 week-pregnancies, *n*)	0	1746	15.4%	898	15.2%	266	17.1%	242	16.0%	135	16.1%	205	13.6%
	1	2062	18.2%	1052	17.8%	302	19.4%	285	18.9%	147	17.5%	276	18.3%
	2	4238	37.4%	2238	37.8%	547	35.2%	579	38.4%	302	35.9%	572	37.9%
	3	2102	18.6%	1117	18.9%	264	17.0%	270	17.9%	165	19.6%	286	18.9%
	4 or more	1180	10.4%	609	10.3%	174	11.2%	133	8.8%	92	10.9%	172	11.4%
Age at menarche, years	Mean/median	13.2/13.0		13.2/13.0		13.2/13.0		13.3/13.0		13.4/13.0		13.2/13.0	
	<12	1369	13.3%	748	13.8%	186	13.5%	163	12.0%	82	11.1%	190	13.8%
	12–13	2104	20.5%	1066	19.7%	308	22.4%	289	21.3%	151	20.4%	290	21.0%
	13–14	2578	25.1%	1359	25.1%	318	23.1%	347	13.3%	188	25.3%	366	26.5%
	>14	4227	41.1%	2249	41.5%	563	40.9%	561	13.3%	321	43.3%	533	38.7%
	Missing	1050		492		178		149		99		132	
Age at FFTP, years	Mean/median	25.1/24.0		25.0/24.0		25.3/25.0		25.6/25.0		25.6/25.0		24.7/24.0	
	<20	822	10.9%	453	11.2%	98	10.5%	89	9.2%	43	8.1%	139	13.2%
	20–25	2948	39.1%	1613	39.8%	353	38.0%	340	35.0%	200	37.8%	442	42.1%
	25–30	2417	32.1%	1292	31.8%	300	32.3%	344	35.4%	184	34.8%	297	28.3%
	>30	1351	17.9%	699	17.2%	179	19.2%	198	20.4%	102	19.3%	173	16.5%
	Missing	2044		1857		623		538		312		460	

*BC* breast cancer, *HER2* human epidermal growth factor receptor-2, *TNBC* triple negative breast cancer, *Pre/peri* premenopausal/perimenopausal, *FFTP* first full-term pregnancy

**Fig. 2:**
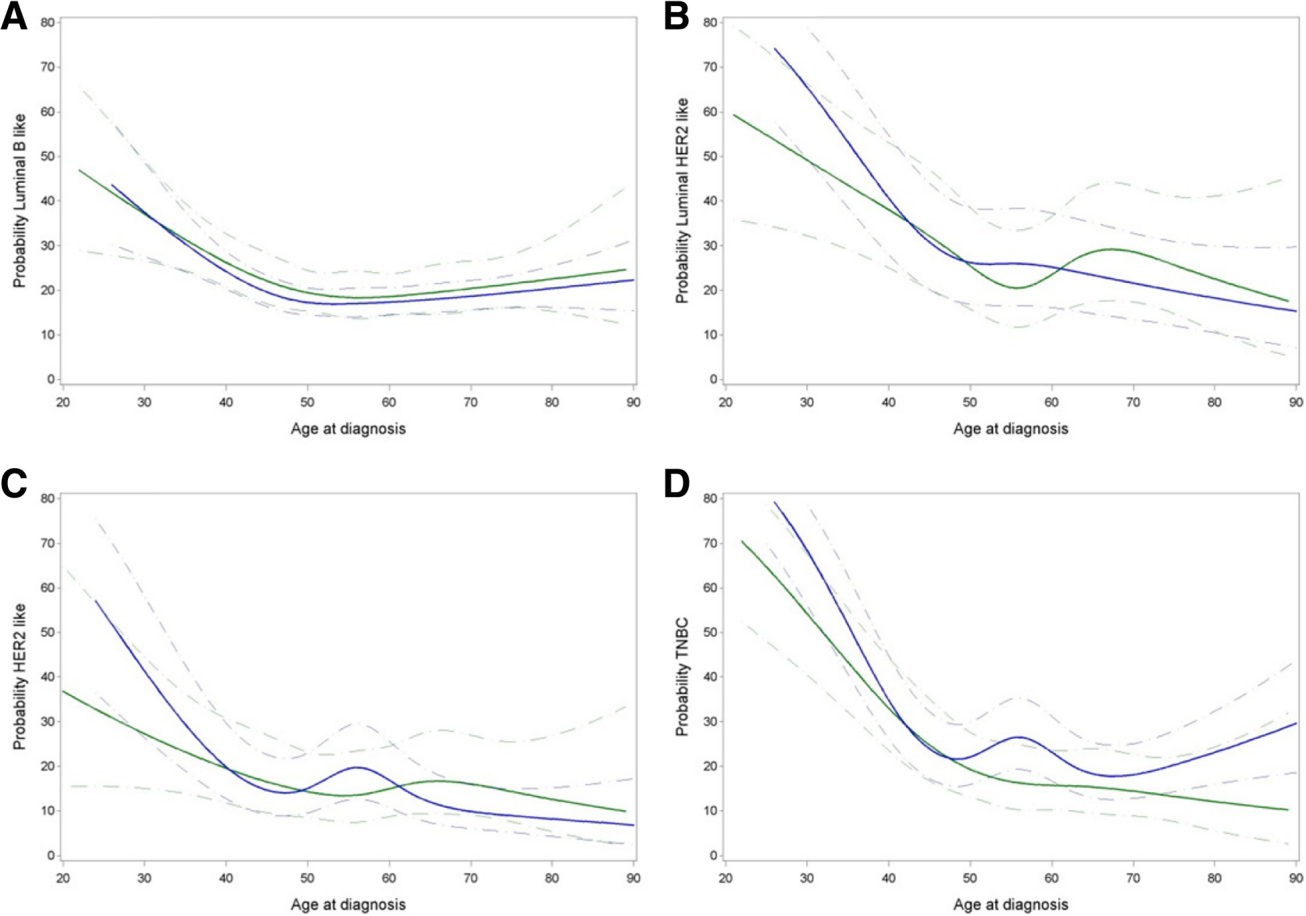
Association between parity and luminal B-like, luminal human epidermal growth factor receptor-2 (HER2)-like, HER2-like and triple-negative breast cancer by age at diagnosis. A binary logistic regression model was fitted considering every molecular subtype as the response variable, while considering luminal A-like breast cancer as a reference category, and parity and age as explanatory continuous variables. Age was modeled non-linearly using cubic splines (5 knots). Blue lines represent probabilities for parous women, green lines probabilities for nulliparous women. **a** Probability of luminal B-like subtype by parity. **b** Probability of Luminal HER2-like subtype by parity. **c** Probability of HER2-like subtype by parity. **d** Probability of triple negative breast cancer (TNBC) subtype by parity

First, we assessed the association between parity and BC subtype compared to luminal A BC as a reference. This was done across all ages modeled non-linearly using cubic splines (see “Methods”), and because of the non-linear fit also extrapolated to specific ages. Table 2 includes specific estimates at 35, 55 and 75 years of age corresponding to premenopausal, early postmenopausal and late postmenopausal ages, whereas Additional file 1: Table S5 highlights estimates at 40, 50 and 60 years of age. A frequency table showing parity by BC subtype and specific age groups is provided as Additional file 1: Table S6. Parous women were more likely to develop TNBC compared to luminal A tumors (OR = 1.38, CI 1.16–1.65, *p* = 0.0004, Table 2), but this association did not vary significantly by age (*p* for interaction = 0.076). Graphical representation of these associations (Fig. 2a-d) nevertheless suggested that parous women were more likely to develop TNBC around age 55 years (Fig. 2d).

**Table 2:** Association between parity (ever versus never) and BC subtypes for age overall and for specific ages (35, 55 and 75 years)

	Age at BC diagnosis	Odds ratio (95% CI)	*P* value	*P* value interaction parity × age
Luminal A-like	All ages	1.00 (Ref.)		
Luminal B-like	All ages	0.90 (0.77–1.05)	0.18	
Luminal HER2-like	All ages	1.04 (0.88–1.24)	0.62	
HER2-like	All ages	1.04 (0.83–1.29)	0.73	
TNBC	All ages	1.38 (1.16–1.65)	0.0004	
Luminal A-like	At 35 years	1.00 (Ref.)		
	At 55 years	1.00 (Ref.)		
	At 75 years	1.00 (Ref.)		
Luminal B-like	At 35 years	0.95 (0.64–1.43)	0.82	
	At 55 years	0.91 (0.65–1.26)	0.55	
	At 75 years	0.89 (0.64–1.24)	0.48	0.99
Luminal HER2-like	At 35 years	1.48 (1.01–2.16)	0.046	
	At 55 years	1.35 (0.92–1.99)	0.13	
	At 75 years	0.72 (0.45–1.14)	0.16	0.037
HER2-like	At 35 years	1.38 (0.86–2.20)	0.18	
	At 55 years	1.56 (0.98–2.47)	0.06	
	At 75 years	0.59 (0.33–1.05)	0.07	0.030
TNBC	At 35 years	1.40 (0.98–2.00)	0.06	
	At 55 years	1.80 (1.24–2.63)	0.002	
	At 75 years	1.67 1.01–2.76)	0.046	0.076

A baseline-category logits model was fitted with breast cancer (BC) subtype as e esponse variable taking luminal A BC as a reference category, and parity and age at diagnosis (as a continuous variable) as explanatory variables. Age was modeled non-linearly using cubic splines (five knots). The *p* value was 0.0149 for interaction effect between parity and age. A random intercept was introduced to account for clustering by study. Interactions between parity and age at BC diagnosis in the probability of developing a specific BC subtype were tested by logistic regression models with binary outcome (1 for the BC subtype and 0 for luminal A-like BC as the reference subtype). The interaction between age and parity in BC subtype risk was tested across all ages and, because age was modeled non-linearly, also specifically at age 35, 55 and 75 years. BC breast cancer, HER2 human epidermal growth factor receptor-2, TNBC triple negative breast cancer

**Fig. 3:**
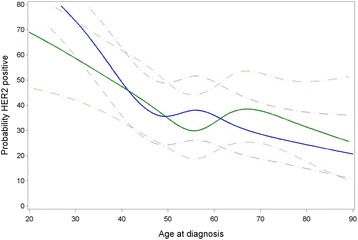
Association between parity and human epidermal growth factor receptor-2 (HER2) + breast cancer by age at diagnosis. A binary logistic regression model was fitted, considering HER2+ breast cancer as the response variable with luminal A-like breast cancer as the reference category. Age was modeled non-linearly using cubic splines (5 knots). Blue lines represent probabilities for parous women, green lines probabilities for nulliparous women

Compared to luminal A tumors, we did not observe a significant association between parity and luminal B-like BC across all ages (OR = 0.90, CI 0.77–1.05, *p* = 0.18), nor at selected ages (Table 2 and Fig. 2a). For luminal HER2-like BC, there was also no significant association with parity across all ages (OR = 1.04, CI 0.88–1.24, *p* = 0.62). We did detect, however, a weak but significant interaction between parity and age (*p* for interaction = 0.037). Indeed, although confidence intervals were wide, parous women were slightly more likely to develop luminal HER2-like BC at age 35 (OR = 1.48, CI 1.01–2.16, *p* = 0.046). This association was not significant in women aged 55 (OR = 1.35, CI 0.92–1.99, *p* = 0.13) and was almost opposite in women aged 75 (OR = 0.72, CI 0.45–1.14, *p* = 0.16, Table 2 and Fig. 2b). Associations between parity and HER2-like breast tumors were similar to those observed for luminal HER2-like BC (Table 2 and Fig. 2c). There was indeed a significant interaction between parity and age (*p* for interaction = 0.030), but at specific ages ORs were not significant. Next, we combined luminal HER2-like and HER2-like BC and investigated whether parity may be associated with the likelihood of developing HER2+ BC (Table 3). A two-way interaction test between parity and age for HER2+ BC relative to luminal A BC was significant (*p* for interaction = 0.003), but a three-way interaction test between parity, age and ER/PR status revealed that the interaction between parity and age does not differ by ER/PR status (*p* for interaction = 0.49). Compared to luminal A BC, parous women were more likely to develop HER2+ BC at age 35 and 55 (OR = 1.44, CI 1.02–2.03, *p* = 0.037 and OR = 1.42, CI 1.04–1.96, *p* = 0.029, respectively), while an inverse association was observed at age 75 (OR = 0.67, CI 0.67–0.98, *P* = 0.041, Table 3). Figure 3 visualizes how the association between parity and HER2+ BC differs by age.

**Table 3:** Association between parity (ever versus never) and HER2+ BC at specific ages (age 35, 55 and 75 years)

	Age at BC diagnosis Odds ratio (95% CI)		*P* value	*P* value interaction parity × age
Luminal A-like	At 35 years	1.00 (Ref.)		
	At 55 years	1.00 (Ref.)		
	At 75 years	1.00 (Ref.)		
HER2+ BC	At 35 years	1.44 (1.02–2.03)	0.037	
	At 55 years	1.42 (1.04–1.96)	0.029	
	At 75 years	0.67 (0.67–0.98)	0.041	0.003

We combined luminal human epidermal growth factor receptor-2 (HER2)-like and HER2-like breast cancer (BC), and investigated whether parity may be associated with the risk of developing HER2+ BC. Interactions between parity and age at BC diagnosis on the probability to develop a specific BC subtype were tested by logistic regression models with binary outcome (1 for HER2+ BC and 0 for luminal A-like BC as the reference subtype) considering parity and age at diagnosis (as a continuous variable) as explanatory variables. A random intercept was introduced to account for clustering by study. The interaction between age and parity on HER2+ BC risk was tested, across all ages and, because age was modeled non-linearly, also specifically at age 35, 55 and 75 years

Next, we assessed whether age at menarche affected the likelihood of being diagnosed with a specific BC subtype, while considering luminal A BC as the reference. The results when parity was considered a dichotomous variable are presented in Table 4, while the data for parity as a continuous variable are presented in Additional file 1: Table S7. For both analyses, we found that in parous and nulliparous women, age at menarche was not significantly associated with any of the subtypes investigated, relative to luminal A BC.

**Table 4:** Associations between age at menarche, age at FFTP and breast cancer subtypes

	Luminal B-like Odds ratio (95% CI)^a^	*P* value	Luminal HER2-like Odds ratio (95% CI)^a^	*P* value	HER2-like Odds ratio (95% CI)^a^	*P* value	TNBC Odds ratio (95% CI)^a^	*P* value
Nulliparous women								
Age at menarche	Linear model		Linear model		Linear model		Linear model	
+ 5 years	1.06 (0.65–1.72)	0.82	1.03 (0.59–1.78)	0.92	1.19 (0.60–2.36)	0.62	1.03 (0.59–1.80)	0.92
Parous women								
Age at menarche	Linear model		Linear model		Linear model		Linear model	
Menarche (+5 years)	1.04 (0.84–1.28)	0.74	0.99 (0.78–1.25)	0.92	1.16 (0.87–1.56)	0.31	0.99 (0.80–1.23)	0.95
Age at FFTP	Linear model (+5 years)		Linear model (+5 years)		Linear model (+5 years)		Quadratic model	
25 versus 20 years							0.78 (0.70–0.88)	<0.0001
30 versus 25 years	1.08 (1.00–1.16)	0.049	1.01 (0.93–1.10)	0.85	1.06 (0.95–1.17)	0.28	0.93 (0.86–1.01)	0.07
35 versus 30 years							1.11 (0.96–1.28)	0.15
Joint analysis age at menarche and age at FFTP								
Age at menarche	Linear model		Linear model		Linear model		Linear model	
Menarche (+5 years)	1.01 (0.79–1.29)	0.92	0.84 (0.64–1.09)	0.19	1.06 (0.76–1.49)	0.71	1.02 (0.80–1.29)	0.90
Age at FFTP	Linear model (+5 years)		Linear model (+5 years)		Linear model (+5 years)		Quadratic model	
25 versus 20 years							0.78 (0.69–0.88)	<0.0001
30 versus 25 years	1.07 (0.99–1.16)	0.10	0.99 (0.91–1.09)	0.91	1.03 (0.92–1.15)	0.62	0.92 (0.85–1.00)	0.045
35 versus 30 years							1.09 (0.94–1.26)	0.27

The results of logistic regression models are reported for each breast cancer (BC) subtype while considering luminal A BC as a reference (the binary response takes values 0 for luminal A or 1 for the subtype that is considered), age at menarche or age at first full-term pregnancy (FFTP) are considered explanatory variables. A random intercept was introduced to account for clustering by study. All analyses reported in this section were performed with correction for age at diagnosis as a continuous variable). Age at menarche or age at FFTP were modeled linearly (for age at menarche), whereas linear and quadratic functions were considered for age at FFTP. The best fit was tested (as described in “Methods”) and are reported here

*HER2* human epidermal growth factor receptor-2, *TNBC* triple negative breast cancer, *CI* confidence interval, *FFTP* first full-term pregnancy

^a^Adjusted for age at diagnosis

Similar analyses were performed for age at FFTP. Increasing age at FFTP was non-linearly associated with the odds of being diagnosed with TNBC relative to luminal A BC. A FFTP at age 25 compared to age 20 years was associated with an OR of 0.78 (CI 0.70–0.88, *p* < 0.0001, Table 4). This association was not apparent for later ages of FFTP. Indeed, patients with a FFTP at age 35 compared to 30 years did not have reduced odds of being diagnosed with TNBC compared to luminal A BC (OR = 1.11, CI 0.96–1.28, *p* = 0.15), whereas TNBC patients with a FFTP at age 30 versus 25 years exhibited an intermediate association (OR = 0.93, CI = 0.86–1.01, *p* = 0.07). For the other BC subtypes, we observed no association with age at FFTP (Table 4). Notably, similar effects were observed when including body mass index (BMI) as a potential confounder in these analyses (Additional file 1: Table S8).

Finally, we also performed a joint analysis to simultaneously assess the association of age at menarche and age at FFTP with BC subtypes (Table 4). Overall, associations were very similar to the associations observed when evaluating age at menarche and age at FFTP separately.

## Discussion

Our analyses in pooled data on 11,328 patients with invasive BC showed that parity is associated with TNBC relative to luminal A disease, irrespective of age at diagnosis. A weak association between parity and luminal HER2-like BC on the other hand could only be observed when assessed at different ages. Furthermore, age at FFTP was non-linearly associated with TNBC.

In an earlier case-only study using BCAC data [3], we reported that parity is associated with a greater probability of being diagnosed with TNBC compared to ER+/HER- or PR+/HER- tumors. In the current analysis, this was confirmed, but relative to luminal A tumors. We did not observe that this association differed significantly according to age (*p* = 0.076), although the effects appeared slightly stronger with older age.

Also in line with previous results reported by the BCAC [3], parity was not associated with HER2-positive BC (both luminal HER2-like and HER2-like BC), while using luminal A-like (HER2-negative) BC as the reference. However, we did observe for the first time that this association differed significantly by age. Parous women diagnosed at 35 years of age were more likely to present with luminal HER2-like or HER2-like rather than luminal A BC (OR = 1.44), whereas parous women diagnosed after the menopause, at 75 years of age, were not (OR = 0.67). We hypothesize that pregnancy may promote HER2 positivity in BCs that are already sub-clinically present during pregnancy, but only become clinically apparent in the years following pregnancy. With older age, we found that parous women are less likely to have HER2-positive BC. Here, pregnancy could induce a protective effect against HER2-positive BCs. Phipps et al. also reported an association between late age at FFTP and increased risk of HER2-like BC, suggesting that there may only be a protective effect of pregnancy against HER2-positive BC when pregnancy precedes carcinogenesis [24].

Several studies already also reported that BC risk varies in the function of the time window between age at menarche and age at FFTP, with most studies suggesting that a short time interval is significantly and inversely associated with ER-positive BC [16, 18, 25, 26]. In the current study, we were able to provide a more detailed analysis of this effect. With respect to age at FFTP, we found that with an older age at FFTP, women were less likely to be diagnosed specifically with TNBC. Interestingly, this association seemed to be stronger for younger ages at FFTP (20–25 years) than for older ages at FFTP (30–35 years). On the other hand, age at menarche was not differentially associated with BC subtypes.

These findings should now be confirmed in large population-based studies. Indeed, the design of our case-only study, in which we calculated odds ratios using luminal A BC as a reference, does not allow us to estimate absolute BC subtype risk. So far, most population-based studies observed a significant inverse association between parity and hormone receptor-positive BC, although none of these studies took age into account [2, 17]. One recent study investigated whether associations between reproductive factors and premenopausal BC differed before and after age 40 years. The inverse association between parity and hormone receptor-positive BC was only observed in women aged > 40 years [27]. Most studies have not identified a statistically significant relationship between parity and the risk of TNBC [2], although one study in women aged between 20 and 44 years observed that a short time interval between menarche and FFTP was associated with an increased risk of TNBC [18]. Again, it should be noted that these studies did not assess differential effects by age or were focused on specific ages of diagnosis, and were often also hampered by the low prevalence of TNBC compared to hormone receptor-positive BC. Possibly, our findings may be explained by Russo’s hypothesis, which suggests that some BCs develop at a younger age (i.e., prior to the pregnancy) [4, 5]. Protection against hormone receptor-positive BC due to a pregnancy may thus result in a relative increase in TNBC at a later age, especially around 55 years, as suggested in this study.

The strength of this study is its large sample size with comprehensive data on molecular markers and other detailed data derived from pathology reports. As such, we were able to derive BC molecular subtypes, which are known to differ in their prognosis, for all included patients. Importantly, we also used clinical criteria based on the 2011 St. Gallen report to refine the definition of the molecular subtypes. We failed, however, to detect obvious differences between luminal B and luminal HER2+ BC with the reproductive factors under study, suggesting that both groups in fact behaved similarly. Our sample size was slightly smaller compared to the previous BCAC study by Yang et al. [3], due to the fact that information on grade had to be available in addition to ER, PR and HER2 status to define subtypes according to the 12th St. Gallen International Breast Cancer Conference Expert Panel [22]. A potential limitation of this study is that data were derived from studies with various designs and methods to obtain risk factor and marker data. Furthermore, a relatively large proportion of patients were diagnosed at a young age, because some participating studies oversampled younger patients with BC and patients with a familial history of BC. In the future, since several new studies have joined BCAC and are in the process of providing more detailed reproductive risk factor data, we plan to take the effect of other variables such as body mass index, age at last pregnancy and breastfeeding into account. However, as we conducted case-case analyses by including a random effect for study in all models and also adjusted for age at diagnosis whenever applicable, it is unlikely that this may have affected our results.

## Conclusion

We report that parity is differentially associated with BC subtypes and that the association for HER2-positive BC (relative to luminal A BC) depends on the patient’s age, but not on ER/PR status. Later age at FFTP was also inversely associated with TNBC, which suggests that an early pregnancy may increase the likelihood of developing TNBC relative to luminal A BC. Our results have to be confirmed in large population-based studies. However, they provide further support for different etiology between BC subtypes and suggest that models used to predict BC risk should take this into account.

## Additional file

Additional file 1: Table S1. Definitions of breast cancer subtypes that have been applied in previous BCAC manuscripts. **Table S2** Number of breast cancer patients with reproductive risk factor data in the 34 BCAC studies assessed in this study. **Table S3** Number of breast cancer case patients with tumor marker data in the 34 BCAC studies assessed in this study. **Table S4** Distribution of tumor characteristics according to breast cancer subtypes. **Table S5** Association between parity (ever versus never) and BC subtypes for age overall and for specific ages (40, 50 and 60 years). **Table S6** Frequency table showing parity by subtype and age group. **Table S7** Associations between age at menarche, age at FFTP and breast cancer subtypes. The same analysis as in Table 4 is performed but here parity is considered a continuous variable. **Table S8** Effect of parity (ever versus never) on BC subtype risk across all ages at BC diagnosis and corrected for BMI. Associations between age at menarche, age at FFTP and breast cancer subtype risk. (DOCX 60 kb)

## Abbreviations

AIC: Akaike information criterion
BC: Breast cancer
BCAC: Breast Cancer Association Consortium
BMI: Body mass index
ER: Estrogen receptor
FFTP: First full-time pregnancy
HER2: Human epidermal growth factor receptor-2
PR: Progesterone receptor
TNBC: Triple negative breast cancer
