# Supplementary material for: Reproductive profiles and risk of breast cancer subtypes: a multi-center case-only study
Source: Breast Cancer Res. 2017 Nov 7;19:119. doi: 10.1186/s13058-017-0909-3 (PMC5688822; doi:10.1186/s13058-017-0909-3)
Supplement: Additional file 1: Table S1. — Definitions of breast cancer subtypes that have been applied in previous BCAC manuscripts. Table S2 Number of breast cancer patients with reproductive risk factor data in the 34 BCAC studies assessed in this study. Table S3 Number of breast cancer case patients with tumor marker data in the 34 BCAC studies assessed in this study. Table S4 Distribution of tumor characteristics according to breast cancer subtypes. Table S5 Association between parity (ever versus never) and BC subtypes for age overall and for specific ages (40, 50 and 60 years). Table S6 Frequency table showing parity by subtype and age group. Table S7 Associations between age at menarche, age at FFTP and breast cancer subtypes. The same analysis as in Table 4 is performed but here parity is considered a continuous variable. Table S8 Effect of parity (ever versus never) on BC subtype risk across all ages at BC diagnosis and corrected for BMI. Associations between age at menarche, age at FFTP and breast cancer subtype risk. (DOCX 60 kb) [file 13058_2017_909_MOESM1_ESM.docx]

**Supplementary Tables**

**Supplementary Table 1:** Definitions of breast cancer subtypes that have been applied in previous BCAC manuscripts.

| **Yang et al. [1] and Broeks et al. [2]** | | **Blows et al. [3]** | **Brouckaert et al. (this study)** |
| --- | --- | --- | --- |
| ER+/HER2- or PR+/HER2- | Grade 1 or Grade 2 | Luminal 1 | Luminal A-like |
|  | Grade 3 |  | Luminal B-like |
| ER+/HER2+ or PR+/HER2+ |  | Luminal 2 | Luminal HER2-like |
| ER-/PR-/HER2+ |  | Non-luminal HER+ | HER2-like |
| ER-/PR-/HER2- |  | TNBC | TNBC |

**Supplementary Table 2:** Number of breast cancer patients with reproductive risk factor data in the 34 BCAC studies assessed in this study.

| **Study Acronym** | **N** | **Study design^a^** | **Age menarche** (mean/median) | **Age FFTP** (mean/median) | **Age breast cancer diagnosis** (mean/median) | **Nulli/(multi-)parous** N (%) |
| --- | --- | --- | --- | --- | --- | --- |
| ABCFS | 621 | Population-based case-control study | 12.8 / 13 | 25.7 / 26 | 39.5 / 38 | 155 (25.0) / 466 (75.0) |
| ABCS | 399 | Mixed design | 13.0 / 13 | 29.4 / 30 | 42.1 / 43 | 114 (28.6) / 285 (71.4) |
| ACP | 124 | Hospital based case-control study | 14.0 / 14 | 25.8 / 25 | 47.0 / 47 | 17 (13.7) / 107 (86.3) |
| BBCC | 482 | Mixed design | 13.5 / 13 | 25.6 / 25 | 60.6 / 62 | 76 (15.8) / 406 (84.2) |
| CECILE | 872 | Population-based case-control study | 12.9 / 13 | 24.7 / 24 | 54.4 / 55 | 95 (10.9) / 777 (89.1) |
| CGPS | 1585 | Mixed design | 13.6 / 14 | 24.8 / 24 | 61.1 / 61 | 212 (13.4) / 1373 (86.6) |
| CNIO-BCS | 210 | Mixed design | 13.2 / 13 | 26.9 / 26 | 55.3 / 55 | 58 (27.6) / 152 (72.4) |
| ESTHER | 386 | Population-based case-control study | 13.6 / 14 | n/a | 60.7 / 62 | 43 (11.1) / 343 (88.9) |
| GENICA | 431 | Population-based case-control study | 13.4 / 13 | 25.4 / 25 | 57.0 / 58 | 77 (17.9) / 354 (82.1) |
| HEBCS | 1041 | Mixed design | 13.3 / 13 | 26.4 / 26 | 57.4 / 56 | 263 (25.3) / 778 (74.7) |
| HERPACC | 442 | Hospital based case-control study | 13.4 / 13 | 26.1 / 26 | 52.4 / 52 | 59 (13.3) / 383 (86.7) |
| KARBAC | 365 | Mixed design | 13.4 / 13 | 25.1 / 25 | 60.7 / 59 | 61 (16.7) / 304 (83.3) |
| KBCP | 367 | Population-based case-control study | 13.8 / 13 | 24.8 / 24 | 59.4 / 57 | 85 (23.2) / 282 (76.8) |
| LAABC | 665 | Population-based case-control study | 12.9 / 13 | n/a | 53.9 / 53 | 167 (25.1) / 498 (74.9) |
| LMBC | 1942 | Mixed design | 13.3 / 13 | 25.5 / 25 | 56.8 / 56 | 282 (14.5) / 1660 (85.5) |
| MARIE | 1561 | Population-based case-control study | 13.5 / 14 | 24.2 / 24 | 62.3 / 63 | 244 (15.6) / 1317 (84.4) |
| MBCSG | 110 | Mixed design | 12.4 / 13 | 28.5 / 28 | 39.4 / 38 | 33 (30.0) / 77 (70.0) |
| MCBCS | 1462 | Mixed design | 12.7 / 13 | 24.2 / 23 | 57.2 / 56 | 178 (12.2) / 1284 (87.8) |
| MCCS | 471 | Prospective cohort | 13.1 / 13 | 25.6 / 25 | 63.5 / 64 | 89 (18.9) / 382 (81.1) |
| MYBRCA | 625 | Mixed design | 13.1 / 13 | 26.4 / 26 | 49.2 / 50 | 117 (18.7) / 508 (81.3) |
| OBCS | 417 | Mixed design | 13.4 / 13 | n/a | 56.3 / 55 | 57 (13.7) / 360 (86.3) |
| OFBCR | 811 | Mixed design | 12.6 / 13 | 24.7 / 25 | 54.1 / 54 | 210 (25.9) / 601 (74.1) |
| ORIGO | 201 | Mixed design | 13.2 / 13 | 26.0 / 26 | 56.3 / 56 | 4 (2.0) / 197 (98.0) |
| RBCS | 366 | Hospital based case-control study | 13.0 / 13 | 26.0 / 25 | 43.7 / 43 | 68 (18.6) / 298 (81.4) |
| SASBAC | 780 | Population-based case-control study | 13.5 / 14 | 25.5 / 25 | 63.1 / 63 | 110 (14.1) / 670 (85.9) |
| SBCGS | 741 | Mixed design | 14.4 / 14 | 27.0 / 27 | 54.8 / 53 | 13 (1.8) / 728 (98.2) |
| SBCS | 447 | Mixed design | 13.1 / 13 | 24.8 / 24 | 61.0 / 61 | 72 (16.1) / 375 (83.9) |
| SEARCH | 4652 | Mixed design | 12.8 / 13 | 24.0 / 23 | 54.3 / 55 | 563 (12.1) / 4089 (87.9) |
| SEBCS | 406 | Mixed design | 15.1 / 15 | n/a | 48.9 / 48 | 21 (5.2) / 385 (94.8) |
| TBCS | 39 | Hospital based case-control study | 14.5 / 15 | 22.4 / 21 | 49.0 / 48 | 0 (0.0) / 39 (100.0) |
| TWBCS | 559 | Hospital based case-control study | 13.9 / 14 | 25.1 / 25 | 51.7 / 51 | 74 (13.2) / 485 (86.8) |
| UKBGS | 106 | Prospective cohort | 12.7 / 13 | 27.3 / 26 | 56.3 / 57 | 11 (10.4) / 95 (89.6) |
| kConFab/AOCS | 153 | Mixed design | 13.0 / 13 | 25.1 / 24 | 45.3 / 44 | 22 (14.4) / 131 (85.6) |
| pKARMA | 4256 | Mixed design | 13.2 / 13 | 25.8 / 25 | 58.6 / 59 | 690 (16.2) / 3566 (83.8) |

^a^ Population-based design was defined as recruiting a random sample of all cases occurring in a geographically defined population during a specified period of time, and recruiting controls that were a random sample of the same source population as cases during the same period of time. Mixed design was defined as not strictly population-based or hospital-based.

n/a = not available

**Supplementary Table 3:** Number of breast cancer case patients with tumor marker data in the 34 BCAC studies assessed in this study.

| **Study Acronym** | **ER-/ER+ N (%)** | **PR-/PR+ N (%)** | **HER2 N (%)** | |
| --- | --- | --- | --- | --- |
| ABCFS | 223 (35.9) / 398 (64.1) | 175 (28.2) / 446 (71.8) | n/a |  |
| ABCS | 106 (26.6) / 293 (73.4) | 169 (42.4) / 230 (57.6) | 227 (57.9) / 165 (42.1) |  |
| ACP | 48 (38.7) / 76 (61.3) | n/a | n/a |  |
| BBCC | 72 (14.9) / 410 (85.1) | 135 (28.0) / 347 (72.0) | 422 (89.6) / 49 (10.4) |  |
| CECILE | 129 (14.8) / 743 (85.2) | 248 (28.8) / 613 (71.2) | 560 (87.0) / 84 (13.0) |  |
| CGPS | 260 (16.4) / 1325 (83.6) | 446 (37.9) / 730 (62.1) | 316 (88.0) / 43 (12.0) |  |
| CNIO-BCS | 53 (25.2) / 157 (74.8) | 87 (42.2) / 119 (57.8) | 61 (65.6) / 32 (34.4) |  |
| ESTHER | 95 (24.6) / 291 (75.4) | 130 (34.4) / 248 (65.6) | 120 (72.7) / 45 (27.3) |  |
| GENICA | 117 (27.1) / 314 (72.9) | 142 (32.9) / 289 (67.1) | 234 (73.6) / 84 (26.4) |  |
| HEBCS | 147 (14.1) / 894 (85.9) | 332 (31.9) / 709 (68.1) | 473 (86.3) / 75 (13.7) |  |
| HERPACC | 121 (27.4) / 321 (72.6) | 169 (38.7) / 268 (61.3) | 183 (76.9) / 55 (23.1) |  |
| KARBAC | 60 (16.4) / 305 (83.6) | 79 (24.8) / 239 (75.2) | n/a |  |
| KBCP | 87 (23.7) / 280 (76.3) | 137 (37.5) / 228 (62.5) | 300 (87.5) / 43 (12.5) |  |
| LAABC | 138 (20.8) / 527 (79.2) | 196 (31.3) / 430 (68.7) | n/a |  |
| LMBC | 306 (15.8) / 1636 (84.2) | 460 (23.7) / 1479 (76.3) | 1573 (85.9) / 258 (14.1) |  |
| MARIE | 353 (22.6) / 1208 (77.4) | 534 (34.2) / 1026 (65.8) | 1133 (80.7) / 271 (19.3) |  |
| MBCSG | 24 (21.8) / 86 (78.2) | 30 (27.3) / 80 (72.7) | 46 (47.9) / 50 (52.1) |  |
| MCBCS | 239 (16.3) / 1223 (83.7) | 372 (25.5) / 1089 (74.5) | 981 (85.0) / 173 (15.0) |  |
| MCCS | 119 (25.3) / 352 (74.7) | 171 (36.3) / 300 (63.7) | 369 (86.2) / 59 (13.8) |  |
| MYBRCA | 254 (40.6) / 371 (59.4) | 237 (48.7) / 250 (51.3) | 281 (50.1) / 280 (49.9) |  |
| OBCS | 81 (19.4) / 336 (80.6) | 119 (28.6) / 297 (71.4) | 361 (86.6) / 56 (13.4) |  |
| OFBCR | 236 (29.1) / 575 (70.9) | 305 (38.5) / 488 (61.5) | n/a |  |
| ORIGO | 47 (23.4) / 154 (76.6) | 60 (35.9) / 107 (64.1) | n/a |  |
| RBCS | 96 (26.2) / 270 (73.8) | 117 (36.9) / 200 (63.1) | 53 (86.9) / 8 (13.1) |  |
| SASBAC | 139 (17.8) / 641 (82.2) | 221 (29.2) / 536 (70.8) | n/a |  |
| SBCGS | 264 (35.6) / 477 (64.4) | 267 (36.1) / 473 (63.9) | n/a |  |
| SBCS | 102 (22.8) / 345 (77.2) | 77 (41.4) / 109 (58.6) | 182 (91.9) / 16 (8.1) |  |
| SEARCH | 906 (19.5) / 3746 (80.5) | 665 (28.6) / 1661 (71.4) | 1668 (88.9) / 209 (11.1) |  |
| SEBCS | 150 (36.9) / 256 (63.1) | 188 (46.3) / 218 (53.7) | 203 (51.3) / 193 (48.7) |  |
| TBCS | 21 (53.8) / 18 (46.2) | n/a | n/a |  |
| TWBCS | 175 (31.3) / 384 (68.7) | 176 (31.6) / 381 (68.4) | 70 (42.4) / 95 (57.6) |  |
| UKBGS | 18 (17.0) / 88 (83.0) | 23 (29.9) / 54 (70.1) | 31 (81.6) / 7 (18.4) |  |
| kConFab/AOCS | 44 (28.8) / 109 (71.2) | 40 (29.9) / 94 (70.1) | n/a |  |
| pKARMA | 668 (15.7) / 3588 (84.3) | 1255 (30.1) / 2921 (69.9) | n/a |  |

n/a = not available

**Supplementary Table 4:** Distribution of tumor characteristics according to breast cancer subtypes.

|  |  | **All breast cancer subtypes** | | **Luminal A-like** | | **Luminal B-like** | | **Luminal HER2-like** | | **HER2-like** | | **TNBC** | |
| --- | --- | --- | --- | --- | --- | --- | --- | --- | --- | --- | --- | --- | --- |
|  |  | **N=11328 (100.0%)** | | **N=5914 (52.2%)** | | **N=1553 (13.7%)** | | **N=1509 (13.3%)** | | **N=841 (7.4%)** | | **N=1511 (13.3%)** | |
|  |  | **N** | **%** | **N** | **%** | **N** | **%** | **N** | **%** | **N** | **%** | **N** | **%** |
| **Tumor size** | pT1 | 4412 | 38.9% | 2746 | 46.4% | 432 | 27.8% | 536 | 35.5% | 241 | 28.7% | 457 | 30.2% |
|  | pT2 | 2714 | 24.0% | 1237 | 20.9% | 522 | 33.6% | 324 | 21.5% | 202 | 24.0% | 429 | 28.4% |
|  | pT3 or pT4 | 761 | 6.7% | 334 | 5.6% | 155 | 10.0% | 91 | 6.0% | 92 | 10.9% | 89 | 5.9% |
|  | pTx | 2689 | 23.7% | 1185 | 20.0% | 336 | 21.6% | 474 | 31.4% | 262 | 31.2% | 432 | 28.6% |
|  | missing | 752 | 6.6% | 412 | 7.0% | 108 | 7.0% | 84 | 5.6% | 44 | 5.2% | 104 | 6.9% |
| **Nodal status** | pN0 | 6148 | 54.3% | 3592 | 60.7% | 734 | 47.3% | 694 | 46.0% | 333 | 39.6% | 795 | 52.6% |
|  | pN+ | 4191 | 37.0% | 1959 | 33.1% | 725 | 46.7% | 602 | 39.9% | 378 | 44.9% | 527 | 34.9% |
|  | pNx | 989 | 8.7% | 363 | 6.1% | 94 | 6.1% | 213 | 14.1% | 130 | 15.5% | 189 | 12.5% |
| **Grade** | G1 | 204 | 1.8% | 1880 | 31.8% | 0 | 0.0% | 111 | 7.4% | 10 | 1.2% | 41 | 2.7% |
|  | G2 | 5136 | 45.3% | 4034 | 68.2% | 0 | 0.0% | 632 | 41.9% | 204 | 24.3% | 266 | 17.6% |
|  | G3 | 3664 | 32.3% | 0 | 0.0% | 1553 | 100.0% | 568 | 37.6% | 505 | 60.0% | 1038 | 68.7% |
|  | Gx | 486 | 4.3% | 0 | 0.0% | 0 | 0.0% | 198 | 13.1% | 122 | 14.5% | 166 | 11.0% |
|  | missing | 1838 | 16.2% | 0 | 0.0% | 0 | 0.0% | 0 | 0.0% | 0 | 0.0% | 0 | 0.0% |
| **PR status** | positive | 3693 | 32.6% | 5068 | 85.7% | 1263 | 81.3% | 1097 | 72.7% | 0 | 0.0% | 0 | 0.0% |
|  | negative | 7428 | 65.6% | 755 | 12.8% | 273 | 17.6% | 372 | 24.7% | 818 | 97.3% | 1475 | 97.6% |
|  | missing | 207 | 1.8% | 91 | 1.5% | 17 | 1.1% | 40 | 2.7% | 23 | 2.7% | 36 | 2.4% |

TNBC: triple negative breast cancer, PR: progesterone receptor

**Supplementary Table 5:** Association between parity (ever versus never) and BC subtypes for age overall and for specific ages (40, 50 and 60 years).

|  | | | **95% confidence interval** | |  |
| --- | --- | --- | --- | --- | --- |
| **Phenotype** | **Diagnosis age** | **Odds Ratio** | **Lower limit** | **Upper Limit** | **P-value** |
| Luminal B like | 40y | 0.90 | 0.67 | 1.20 | 0.4674 |
| Luminal B like | 50y | 0.86 | 0.67 | 1.11 | 0.2555 |
| Luminal B like | 60y | 0.92 | 0.70 | 1.20 | 0.5284 |
| Luminal HER2 like | 40y | 1.11 | 0.82 | 1.49 | 0.5009 |
| Luminal HER2 like | 50y | 1.04 | 0.79 | 1.36 | 0.7780 |
| Luminal HER2 like | 60y | 1.10 | 0.80 | 1.50 | 0.5615 |
| HER2 like | 40y | 1.01 | 0.70 | 1.47 | 0.9512 |
| HER2 like | 50y | 1.06 | 0.75 | 1.49 | 0.7393 |
| HER2 like | 60y | 1.16 | 0.80 | 1.68 | 0.4438 |
| TNBC | 40y | 1.07 | 0.79 | 1.45 | 0.6395 |
| TNBC | 50y | 1.18 | 0.90 | 1.56 | 0.2304 |
| TNBC | 60y | 1.61 | 1.17 | 2.22 | 0.0031 |
|  | | | | | |

**Supplementary Table S6:** Frequency table showing parity by subtype and age group.

|  | | **Nulliparous** | | **Parous** | |
| --- | --- | --- | --- | --- | --- |
| **Age** | **BC subtype** | **N** | **%** | **N** | **%** |
|  |  |  |  |  |  |
| <41y | Luminal A like | 89 | 5.1 | 239 | 2.5 |
| <41y | Luminal B like | 45 | 2.6 | 94 | 1.0 |
| <41y | Luminal HER2 like | 61 | 3.5 | 186 | 1.9 |
| <41y | HER2 like | 29 | 1.7 | 91 | 1.0 |
| <41y | TNBC | 66 | 3.8 | 177 | 1.8 |
|  |  |  |  |  |  |
| 41-50y | Luminal A like | 220 | 12.6 | 1149 | 12.0 |
| 41-50y | Luminal B like | 70 | 4.0 | 297 | 3.1 |
| 41-50y | Luminal HER2 like | 72 | 4.1 | 383 | 4.0 |
| 41-50y | HER2 like | 37 | 2.1 | 189 | 2.0 |
| 41-50y | TNBC | 53 | 3.0 | 289 | 3.0 |
|  |  |  |  |  |  |
| 51-60y | Luminal A like | 278 | 15.9 | 1564 | 16.3 |
| 51-60y | Luminal B like | 65 | 3.7 | 359 | 3.7 |
| 51-60y | Luminal HER2 like | 51 | 2.9 | 355 | 3.7 |
| 51-60y | HER2 like | 36 | 2.1 | 251 | 2.6 |
| 51-60y | TNBC | 45 | 2.6 | 436 | 4.6 |
|  |  |  |  |  |  |
| >60 | Luminal A like | 313 | 17.9 | 2062 | 21.5 |
| >60 | Luminal B like | 86 | 4.9 | 537 | 5.6 |
| >60 | Luminal HER2 like | 60 | 3.4 | 341 | 3.6 |
| >60 | HER2 like | 34 | 1.9 | 174 | 1.8 |
| >60 | TNBC | 42 | 2.4 | 403 | 4.2 |
| % of Phenotype*age subgroup among all (Nulli)parous | | | | | |

**Supplementary Table S7:** Associations between age at menarche, age at FFTP and breast cancer subtypes. The same analysis as in Table 4 is performed but here parity is considered a continuous variable.

|  | | | **95% confidence interval** | |  |
| --- | --- | --- | --- | --- | --- |
| **BC Subtype** |  | **Odds Ratio** | **Lower Limit** | **Upper Limit** | **P-value** |
| Luminal B like | Age menarche (5y increase) | 1.01 | 0.79 | 1.29 | 0.9212 |
| Luminal B like | Age FFTP (5y increase) | 1.07 | 0.98 | 1.16 | 0.1156 |
| Luminal B like | Parity (+1 pregnancy) | 1.00 | 0.93 | 1.08 | 0.9647 |
| Luminal HER2 like | Age menarche (5y increase) | 0.84 | 0.64 | 1.09 | 0.1931 |
| Luminal HER2 like | Age FFTP (5y increase) | 0.99 | 0.90 | 1.08 | 0.7841 |
| Luminal HER2 like | Parity (+1 pregnancy) | 0.98 | 0.90 | 1.06 | 0.5710 |
| HER2 like | Age menarche (5y increase) | 1.07 | 0.76 | 1.49 | 0.7020 |
| HER2 like | Age FFTP (5y increase) | 1.02 | 0.91 | 1.14 | 0.7440 |
| HER2 like | Parity (+1 pregnancy) | 0.98 | 0.88 | 1.09 | 0.6596 |
| TNBC | Age menarche (5y increase) | 1.02 | 0.80 | 1.30 | 0.8949 |
| TNBC | Age FFTP (20y vs 25y) | 0.78 | 0.69 | 0.88 | <.0001 |
| TNBC | Age FFTP (25y vs 30y) | 0.92 | 0.84 | 1.00 | 0.0484 |
| TNBC | Age FFTP (30y vs 35y) | 1.08 | 0.93 | 1.26 | 0.2923 |
| TNBC | Parity (+1 pregnancy) | 0.99 | 0.92 | 1.07 | 0.8375 |
| HER2 positive | Age menarche (5y increase) | 1.07 | 0.76 | 1.49 | 0.7020 |
| HER2 positive | Age FFTP (5y increase) | 1.02 | 0.91 | 1.14 | 0.7440 |
| HER2 positive | Parity (+1 pregnancy) | 0.98 | 0.88 | 1.09 | 0.6596 |
| Age menarche: Linear model in all cases. Age FFTP: Linear model for Luminal B-like, Luminal HER2-like, HER2-like, quadratic model for TNBC. Parity: Linear model in all cases. | | | | | |

**Supplementary Table S8:** Effect of parity (ever versus never) on BC subtype risk across all ages at BC diagnosis and corrected for BMI. Associations between age at menarche, age at FFTP and breast cancer subtype risk. We present the same analyses as shown in Table 2 and 4 of the main manuscript, but here also correct for body-mass index (BMI). These analyses were performed separately because 12.9% of BMI data were missing.

**Effect of parity on subtype BC risk (corrected for BMI)**

|  | | **95% confidence interval** | |  |
| --- | --- | --- | --- | --- |
| **Phenotype** | **Odds Ratio** | **Lower limit** | **Upper Limit** | **P-value** |
| Luminal B like | 0.86 | 0.73 | 1.01 | 0.0690 |
| Luminal HER2 like | 1.05 | 0.87 | 1.27 | 0.6355 |
| HER2 like | 1.09 | 0.85 | 1.38 | 0.5086 |
| TNBC | 1.42 | 1.17 | 1.72 | 0.0004 |
| Results based on separate logistic regression models with binary outcomes: e.g. Luminal B-like vs Luminal A-like, etc. | | | | |

**Associations between age at menarche, age at FFTP and breast cancer subtypes**

**(corrected for BMI)**

|  | **Luminal B-like** |  | **Luminal HER2-like** | | **HER2-like** |  | **TNBC** |  |
| --- | --- | --- | --- | --- | --- | --- | --- | --- |
|  | **Odds ratio (95% CI)^a^** | **p-value** | **Odds ratio (95% CI)^a^** | **p-value** | **Odds ratio (95% CI)^a^** | **p-value** | **Odds ratio (95% CI)^a^** | **p-value** |
| **Nulliparous Women** |  | |  | |  | |  | |
| **Age menarche** | **Linear model** |  | **Linear model** | | **Linear model** | | **Linear model** | |
| +5y | 1.11 (0.89-1.39) | 0,36 | 1.03 (0.80-1.32) | 0,83 | 1.24 (0.91-1.70) | 0,18 | 1.08 (0.86-1.36) | 0,51 |
| **Parous Women** |  | |  | |  | |  | |
| **Age menarche** | **Linear model** |  | **Linear model** | | **Linear model** | | **Linear model** | |
| Menarche (+5y) | 1.20 (0.72-2.01) | 0.49 | 1.10 (0.61-1.97) | 0.75 | 1.08 (0.51-2.30) | 0.83 | 1.10 (0.60-1.98) | 0,76 |
| **Age FFTP** | **Linear model (+5y)** | | **Linear model (+5y)** | | **Linear model (+5y)** | | **Quadratic model** | |
| 25 versus 20y | 1.09 (1.00-1.17) | 0,044 | 0.99 (0.90-1.08) | 0,77 | 1.06 (0.94-1.18) | 0,35 | 0.77 (0.68-0.87) | <.0001 |
| 30 versus 25y |  |  |  |  |  |  | 0.91 (0.84-0.99) | 0,04 |
| 35 versus 30y |  |  |  |  |  |  | 1.08 (0.93-1.26) | 0,32 |

**References**

1. Yang, X.R., et al., *Associations of breast cancer risk factors with tumor subtypes: a pooled analysis from the Breast Cancer Association Consortium studies.* J Natl Cancer Inst, 2011. **103**(3): p. 250-63.

2. Broeks, A., et al., *Low penetrance breast cancer susceptibility loci are associated with specific breast tumor subtypes: findings from the Breast Cancer Association Consortium.* Hum Mol Genet, 2011. **20**(16): p. 3289-303.

3. Blows, F.M., et al., *Subtyping of breast cancer by immunohistochemistry to investigate a relationship between subtype and short and long term survival: a collaborative analysis of data for 10,159 cases from 12 studies.* PLoS Med, 2010. **7**(5): p. e1000279.
